# Increased sagittal diameter of the vertebral arch aids in diagnosis of lumbar spondylolysis

**DOI:** 10.1007/s00256-020-03658-8

**Published:** 2020-10-28

**Authors:** Shi-Zheng Chen, An-Ni Tong, He-Hu Tang, Zhen Lv, Shu-Jia Liu, Jie-Sheng Liu, Jun-Wei Zhang

**Affiliations:** 1 Department of Spine and Spinal Cord Surgery, China Rehabilitation Research Center, Beijing, China; 2 Department of Orthopedic Surgery, Capital Medical University, Beijing, China; 3 Faculty of Rehabilitation Medicine, Capital Medical University, Beijing, China

**Keywords:** Spondylolysis, Lumbar vertebrae, X-rays

## Abstract

**Objective:**

To identify a diagnostic indicator of lumbar spondylolysis visible in plain X-ray films.

**Methods:**

One hundred and seventy-two patients with low back pain who received X-ray and computerized tomography (CT) examinations were identified and studied. They were divided into three groups: the spondylosis without spondylolisthesis (SWS) group, comprising 67 patients with bilateral pars interarticularis defects at L5 and without spondylolisthesis, the isthmic spondylolisthesis (IS) group, comprising 74 patients with L5/S1 spondylolisthesis and bilateral L5 pars interarticularis defects, and the control group, comprising 31 patients with low back pain but without spondylolysis. The sagittal diameters of the vertebral arch (SDVAs) of L4 and L5 were measured in lateral X-ray image, and the differences in SDVA between L4 and L5 (DSL4-5) in each case were calculated and analyzed.

**Results:**

There were no significant differences in demographic characteristics among the three groups. In the SWS and IS groups, the SDVA of L5 was significantly longer than the SDVA of L4 (*p <* 0.001), whereas no significant difference found in the control group (*p* > 0.05). DSL4-5, in which the SDVA of L4 was subtracted from the SDVA of L5, significantly differed among the three groups (*p* < 0.001), and the normal threshold was provisionally determined to be 1.55 mm.

**Conclusions:**

In bilateral L5 spondylolysis, the SDVA of L5 is wider than the SDVA of L4, and this difference is greater in isthmic spondylolisthesis. This sign in lateral X-rays may provide a simple and convenient aid for the diagnosis of spondylolysis.

## Introduction

Spondylolysis, derived from the Greek words spondylos (vertebra) and lysis (defect) [1–3], is defined as a unilateral or bilateral defect or abnormality of the pars interarticularis and surrounding lamina and pedicle. Approximately 80% of patients with spondylolysis are asymptomatic, and the defect is found incidentally. Spondylolysis can be a cause of low back pain (LBP) in children, adolescents, and adults, arising on hyperextension and worsening with activity [4]. In clinical practice, a considerable number of people with LBP receive outpatient treatment. Among them, patients with spondylolysis complain of hamstring tightness and/or neurologic symptoms such as occasional pain radiating to the buttocks or proximal lower extremities [5]. After physical examination, physicians often order a lumbar X-ray as the primary imaging test. This test may provide direct or indirect evidence of structural lesions in the lumbar spine. If a deformity is found in the X-rays, such as narrowing of the intervertebral space, spondylolysis, or osteoporosis, a diagnosis can be made. Unfortunately, these signs may be absent, and pars interarticularis defects (PID) are often difficult to see in X-rays, especially if there is no spondylolisthesis, in which case the patients are likely to be scheduled for further imaging, such as CT and MRI. However, in some facilities, no such devices may be available, or these tests may not be possible for cost-effectiveness reasons. Therefore, physicians are advised to review the plain X-ray film again, particularly the lateral view, because a sign of lumbar spondylolysis may be present on plain X-ray film. The aim of our study was to identify a diagnostic indicator of lumbar spondylolysis visible on plain X-ray films.

## Materials and methods

### Study participants

Clinical materials, including X-rays and CT images of patients with LBP who visited our outpatient department between February 2012 and December 2017, were collected and reviewed. The inclusion criteria required the outline of the posterior wall and the spinous process of the L4 and L5 vertebrae to be accurately identifiable, and spondylolysis was diagnosed by using CT scans. Cases with vertebral fracture, spinal deformity, tumor, infection, rheumatism, metabolic disease, or radiographs of poor quality were excluded. One hundred and seventy-two patients were included and divided into three groups. Sixty-seven patients (36 males and 31 females; age, median ± interquartile range, 47.0 ± 17.0 years) with bilateral pars interarticularis defects (PID) only at L5, but without spondylolisthesis, were placed in the spondylosis without spondylolisthesis (SWS) group; 74 patients (38 males and 36 females; age: 54.0 ± 18.5 years) with L5/S1 spondylolisthesis and bilateral L5 PID were placed in the isthmic spondylolisthesis (IS) group and 31 patients (19 males and 12 females, age: 58.0 ± 26.0 years) with LBP but no abnormalities in lumbar spine images were classified as the control group (Table 1).

**Table 1:** Characteristics of patients with spondylolysis and normal participants

Group	*n*	Sex		Age (year)	SDVA (mm)		DSL4-5 (mm)
		Male	Female		L4	L5	
SWS	67	36	31	47.0 ± 17.0	22.76 ± 3.61	28.08 ± 9.70	5.60 ± 9.00
IS	74	38	36	54.0 ± 18.5	22.84 ± 2.69	34.37 ± 7.07	10.80 ± 6.15
Control	31	19	12	58.0 ± 26.0	23.62 ± 1.87	23.45 ± 2.18	0.10 ± 0.50
*P*		*p* > 0.05 (*p* = 0.646)		*p*>0.05 (*p* = 0.059)	*p* > 0.05 (*p* = 0.176)	*p* < 0.05 (*p* < 0.001)	*p* < 0.05 (*p* < 0.001)

Continuous variables are presented as median ± interquartile range

*SWS* spondylolysis without spondylolisthesis, *IS* isthmic spondylolisthesis, *SDVA* sagittal diameter of the vertebral arch, *DSL4-5* difference between the SDVAs of L4 and L5, calculated by subtracting the SDVA of L4 from the SDVA of L5

### Radiological assessment

**Fig. 1:**
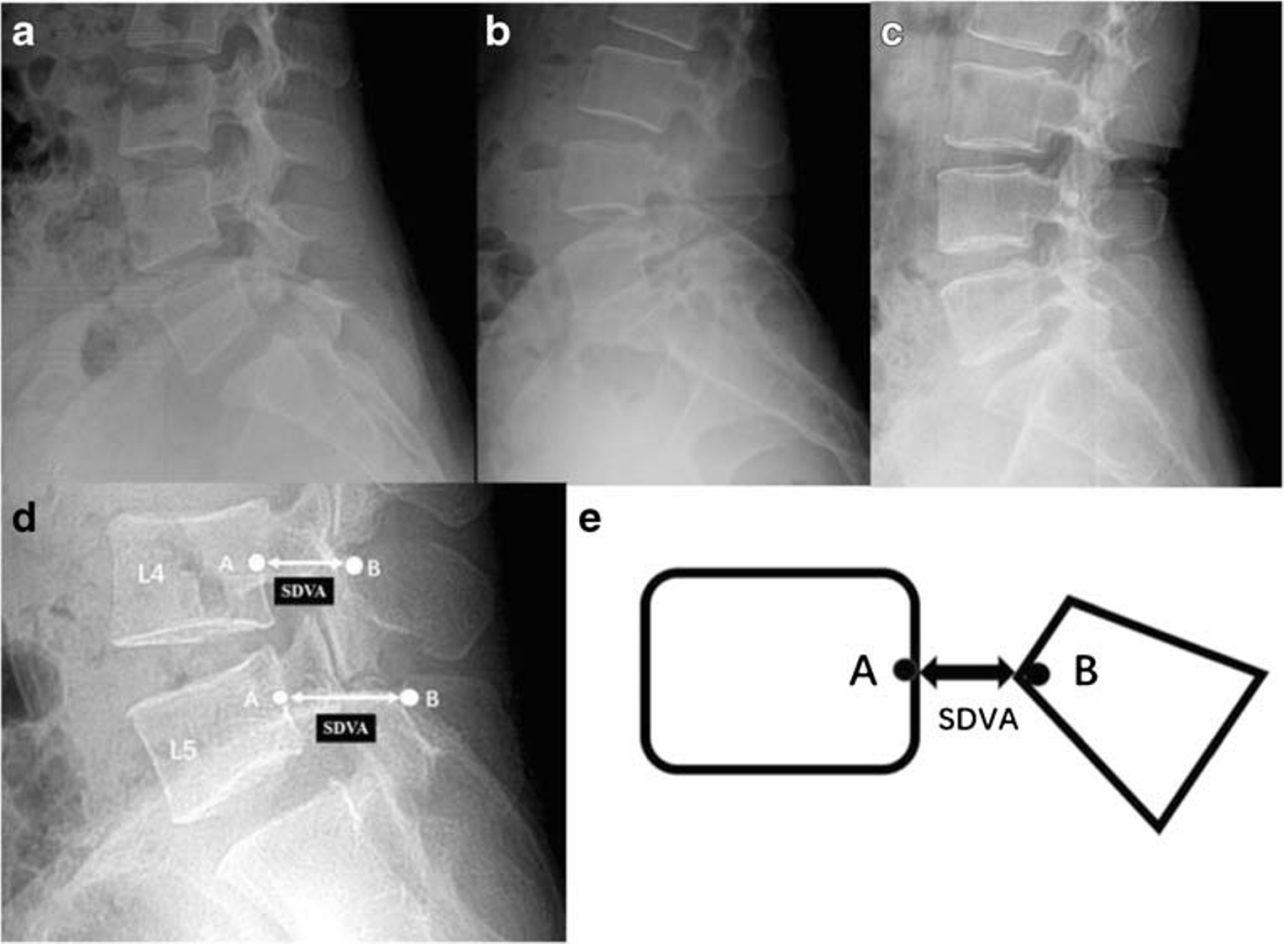
Images of the spondylolysis and control groups, and measurement of the sagittal diameter of the vertebral arch (SDVA) on radiographs. **a** X-ray from the spondylosis without spondylolisthesis (SWS) group. **b** X-ray from the isthmic spondylolisthesis (IS) group; **c** X-ray from the control group. **d**, **e** Measurement of the sagittal diameter of the vertebral arch (SDVA). A: the midpoint of the posterior vertebral wall, B: the point at the root of the corresponding spinous process, on the outer line of the lamina, closest to point A

**Fig. 2:**
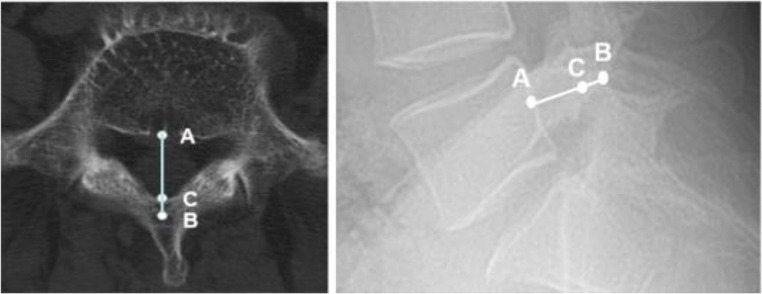
Illustration of key points in SDVA measurement. Selecting B as the closest point to A on the ventral side of the spinous cancellous bone. AC represents the median sagittal diameter of the spinal canal

Synapse 3.2.1 (FUJIFILM MEDICAL SYSTEM, USA, Inc.) software was used to view and measure the images. First, the plain X-ray and CT images were carefully scrutinized to confirm the diagnosis and grouping of the patients. Then in the lateral view of the lumbar X-rays, for L4 or L5, the midpoint of the posterior vertebral wall was marked as A. The point at the root of the corresponding spinous process, on the outer line of the lamina and closest to point A, was marked as B. The AB distance was defined as the sagittal diameter of the vertebral arch (SDVA). Care was taken to select point B, which is the closest point to A on the ventral side of the spinous cancellous bone, rather than point C on the ventral side of the lamina. AC is well known to represent the median sagittal diameter of the spinal canal. However, in such images, point C is usually difficult to find, whereas point B is much clearer (Figs. 1 and 2). For each participant, the SDVAs of L4 and L5 were measured, and the differences between the SDVAs of L4 and L5 were calculated by subtracting the SDVA of L4 from the SDVA of L5; this difference is denoted DSL4-5. Two physicians measured the film for each patient separately, and the mean of the two measurements was used as the value in each case. To demonstrate the contribution of DSL4-5 to the diagnosis of spondylolysis, we reviewed all the images once more, then divided the patients into two groups on the basis of L4 bilateral PID being directly seen or not seen on the plain X-ray films.

### Statistical analysis

The intraclass coefficient was used to analyze the repeatability between the two physicians’ measurements. Pearson’s Chi-square test was applied to compare the sex of the participants, and the Kruskal-Wallis test was used to compare the age, SDVA-L4, SDVA-L5, and DSL4-5 among groups. Wilcoxon rank sum test was used to investigate the differences in the SDVA of L4 and the SDVA of L5 among groups. ROC curve analysis was performed to assess the accuracy of the new measurement method and to provide a possible threshold. *P* < 0.05 was considered statistically significant.

## Results

There was no difference in sex and age among the three groups (*p* > 0.05; Table 1).

In the SWS, IS, and control groups, the average SDVA of L4 was 22.76 ± 3.61 mm, 22.84 ± 2.69 mm, and 23.62 ± 1.87 mm, respectively, whereas that of L5 was 28.08 ± 9.70 mm, 34.37 ± 7.07 mm, and 23.45 ± 2.18 mm, respectively, and the DSL4-5 was 5.60 ± 9.00 mm, 10.80 ± 6.15 mm, and 0.10 ± 0.50 mm, respectively (Table 1). There were statistically significant differences in the SDVA of L5 and DSL4-5 among the three groups (*p* < 0.001), but no significant difference in the SDVA of L4 (*p* > 0.05). Regarding intra group differences, the L4 and L5 difference in the IS group was largest, followed by the SWS group, and there was no significant difference between L4 and L5 in the control group.

**Fig. 3:**
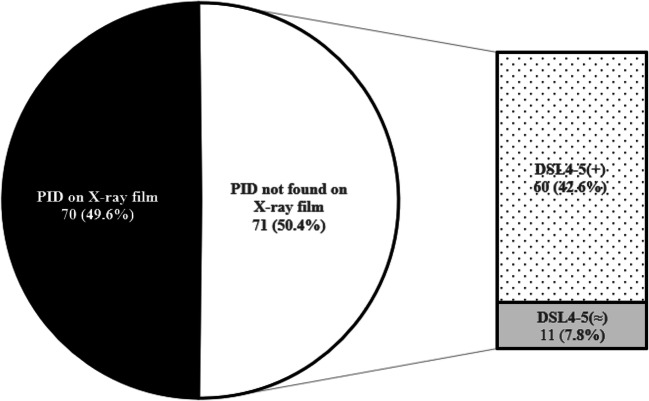
Diagnosis of spondylolysis by using plain X-ray film and DSL4-5; 85% of the patients with no direct evidence of PID on the lateral view showed abnormal DSL4-5, which led to the diagnosis of spondylolysis

Seventy (49.6%) of 141 patients in the SWS and IS groups were able to be diagnosed by direct evidence of PID on the plain X-ray radiographs. Among the remaining 71 cases, 60 were found to have a significant enlargement of DSL4-5 (Fig. 3).

**Fig. 4:**
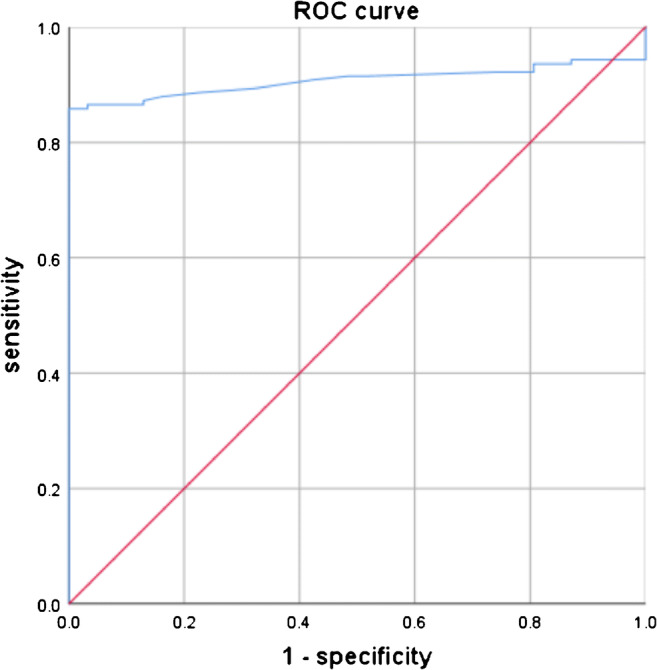
ROC curve of the DSL4-5 diagnosis model of spondylolysis. The large area under the ROC curve indicates the high accuracy of our method

The inter-observer reliability of the image measurements was high (ICC = 0.966, CI: 0.955–0.975), and the area under the ROC curve was 0.907 (Fig. 4). The cut-off value found by using the Youden index showed high sensitivity (0.858) and specificity (1.0). The threshold of DSL4-5 was determined to be 1.55 mm by analysis of the ROC curve. One hundred and twenty-one cases (85.8%) in all 141 patients with spondylolysis and 50 patients (74.6%) in the SWS group were able to be diagnosed with PID by using DLS4-5 and this threshold.

## Discussion

Approximately 25% of patients with lumbar spondylolysis have low back pain or sciatica because of muscular and ligamentous strain, facet degeneration, spinal or foraminal stenosis, and associated disk degeneration or herniation [6, 7]. Isthmic spondylolysis is multifactorial in nature. Masharawi has grouped the factors into four clusters: anatomic, according to the size and shape of the vertebrae [8]; developmental, congenital, or hereditary [9]; spinal alignment [10]; and activity, stress intensity, and movement [10, 11]. The determinants of whether lumbar spondylolysis will develop into LBP or spondylolisthesis are not well understood. Early detection and therapy for isthmus defects may prevent the progression of spondylolysis, and helping patients avoid surgical interventions such as arthrodesis or direct pars repair is important [12].

Diagnosis of spondylolysis is mainly established by imaging of the spine, such as through X-ray, CT, single-photon emission computed tomography (SPECT), or MRI. The most common examination is X-ray imaging. Amato et al. have reported that diagnosing spondylolysis by X-ray anterolateral and bilateral oblique films is sufficient, with a diagnosis rate of 96.5% on the basis of five views (anteroposterior, lateral, 45° right and left oblique views, and collimated lateral views) [13]. The “collar” of a “Scottish dog” which represents lysis in the pars on the oblique radiographic views of the spine is thought to constitute evidence for diagnosis. Plain films are also useful to detect other anomalies of the vertebra and surrounding structures, such as spondylolisthesis, scoliosis, and spina bifida [14]. CT allows for visualization of more details of vertebral morphology and has been found to be the most accurate method for detecting the course and the extent of pars defects, with a sensitivity of 90% [15–17]. MRI, which causes little harm because of the lack of ionizing radiation, is a valuable tool for early diagnosis of spondylolytic lesions and for patients with neurological deficits. Although CT, SPECT, and MRI may provide greater diagnostic accuracy, some hospitals are unable to complete these examinations because of a lack of imaging equipment. Furthermore, CT and SPECT should be carefully used, particularly in children and adolescents, because of radiation exposure [15]. Because a plain X-ray film was usually already available before we decided to use CT to confirm our assessments, scrutinizing the film was a good alternative to ordering a CT or MRI.

Plain film is not only the first choice in initial diagnosis but can be easily performed by almost every hospital or clinic, thus greatly reducing the equipment requirements. Miller et al. have noted that there is no significant difference between AP and lateral views versus additional oblique views in the sensitivity of spondylolysis diagnosis (78% vs. 72%, *p* = 0.39) [15]. Therefore, this method enables the most economical use of medical resources; making the diagnosis on the basis of only by AP and lateral radiographs is also safest and most ideal for patients. Though Amato et al. have reported that 80% of spondylolysis cases can be observed by AP and lateral radiography [13], other studies have improved the diagnose rate by measuring on AP or lateral view. For example, G. Ravichandran has reported that using the position of adjacent spinous processes on AP radiographs can be used to diagnose spondylolysis, because its spinous processes may deviate from the midline or rotate [18]. D. Bryk et al. have reported a spinous process step-off sign, in which the process sequence is no longer smooth, and there is a step-like change in lateral view [19]. H. Saraste et al. have reported that the ratio of posterior to anterior height of L5 vertebral body is smaller in spondylolysis and spondylolisthesis [20]. Jin Yin et al. have suggested that an increase in pelvic incidence (PI) and a decrease in the sacral table angle (STA) might indicate spondylolysis [21]. Our study provides a simple diagnostic method from lateral plain film; this method is similar to the wide-canal sign in MRI [22]. The use of DSL4-5 may facilitate rapid diagnosis of spondylolysis.

According to clinical anatomy studies, greater inter-facet distance and lordosis [8], more frontally oriented lower lumbar facets [9], wider and longer vertebrae body dimensions, a relatively longer spinous process, longer isthmus and lamina, a longer and larger vertebral canal (“spinous process step-off sign” [19] or “wide-canal sign” [22]) and a more lordotic vertebrae body [9, 11] can be found in spondylolysis. In particular, an elongated isthmus [10] and isolated posterior element subluxation [11] in spondylolysis may explain why the SDVA of L5 was longer than that of L4 in our study. The absolute value of SDVA was not recommended as a diagnostic criterion because it was likely to be influenced by factors such as race, age, BMI, and sex. DSL4-5, the difference between the adjacent segments within an individual, which effectively avoids the influence of the abovementioned factors, was thus used in the present study.

The normal resolution of the human eye is 1 arc min, which means that a physician can distinguish two points 0.1 mm apart on a screen 30 cm away [23]. Hence, the difference of 1.55 mm can easily be recognized by the naked eye. In this study, though only 49.6% of SWS and IS cases could be diagnosed by observation of the isthmus on lateral radiographs, the diagnostic efficiency was then improved by examination of DSL4-5 (Fig. 4). Therefore, relatively accurate diagnosis of lumbar spondylolysis and isthmic spondylolisthesis could be achieved by using a single plain X-ray image. The diagnostic procedure was simple and did not require complex imaging examinations or calculations. The emphasis of this study was on how to improve the diagnosis of lumbar spondylosis when only a plain A-P and lateral views of X-ray are taken, as is often done in most hospitals or clinics. The “Scottish dog” sign may have high accuracy in diagnosing spondylolysis but requires an oblique film to additionally be taken. Thus, the two signs could not be compared directly. However, such a comparison would be an interesting topic worthy of further study.

In summary, in patients with bilateral spondylolysis at L5, the SDVA was greater in L5 than L4, and this difference was easily identifiable in lateral X-ray views. This finding indicated the separation of the spinous process and vertebral body, as if they are saying good bye to each other. This sign may provide a simple and convenient way to diagnose spondylolysis.

## Abbreviations

L4: Lumbar 4
SDVA: Sagittal diameter of the vertebral arch
DSL4-5: Difference in SDVA between L4 and L5, calculated by subtracting SDVA of L4 from the SDVA of L5
PID: Pars interarticularis defect
IS: Isthmic spondylolisthesis
SWS: Spondylosis without spondylolisthesis
LBP: Low back pain
CT: Computerized tomography
